# The magnitude of hypertension and its risk factors in southern Ethiopia: A community based study

**DOI:** 10.1371/journal.pone.0221726

**Published:** 2019-08-28

**Authors:** Alemayehu Zekewos, Tariku Egeno, Eskindir Loha

**Affiliations:** 1 Hawassa University, College of Medicine and Health Sciences, Biochemistry Unit, Hawassa, Ethiopia; 2 Hawassa University, College of Medicine and Health Sciences, Department of Internal Medicine, Hawassa, Ethiopia; 3 Hawassa University, College of Medicine and Health Sciences, School of Public Health, Hawassa, Ethiopia; Edith Cowan University, AUSTRALIA

## Abstract

**Background:**

Prevention and control of hypertension has not been given due attention though previous studies indicated that hypertension is growing public health problem.

**Objective:**

This study aimed to determine the prevalence of hypertension and associated factors in Bona district, southern Ethiopia.

**Methods:**

A community based cross-sectional study was conducted on 1952 participants aged ≥25 years in Bona District, southern Ethiopia. Data were collected from consented participants recruited using multistage sampling technique. Data were entered, checked for quality and analyzed by SPSS for Windows version 20.0. Since the outcome variables were ordered categorical, we used multinomial logistic regression model to identify associated factors. Among the independent variables included in the model no multicolinearity was observed. The level of significance was set at P value ≤ 0.05.

**Results:**

The observed prevalence of hypertension (21.8%) was remarkable in rural setting. Out of hypertensive participants, 195 (45.9%) were newly diagnosed. About one third of the participants (31.4%) had central obesity measured by waist-to-height ratio ≥0.50. Being male, age advancement, high BMI (≥25.0 kg/m^2^) and central obesity (waist-to-height ratio ≥0.50) were positively associated with both systolic and diastolic hypertension. Systolic hypertension was negatively associated with high family income. The likely hood of developing diastolic hypertension increased in participants with family history of hypertension.

**Conclusion:**

The overall prevalence of hypertension, 21.8%, is alarmingly high that it can be said that hypertension is becoming a silent epidemic in Ethiopia. Nationwide survey is needed to get the clear magnitude of hypertension so that early detection and management strategies can be enforced.

## Introduction

Hypertension is an important determinant of cardiovascular disease (CVD) and mortality, and accounts for 7.5 million (12.8%) of deaths per year [1,2]. Risk Assessment Collaborating group has identified hypertension as the leading global risk factor for mortality and the third leading risk factor for disease burden [2,3]. The incidence of hypertension is increasing globally due to current nutritional transition, sedentary lifestyle, excessive body weight and other modifiable risk factors. Different studies have indicated that it is increasing drastically in recent years in developing countries while it remained stable and decreased in developed countries [4]. Over 25% of world’s adult population was affected by hypertension in 2000 and projected to raise to 30% in 2025 [2].

In Africa, the national prevalence of hypertension in age group of 25–65 years ranges from 25% to 35% and it remains the most important contributor for increased mortality from cardiovascular diseases [5,6]. Kearaney et al estimated that hypertension affected 639 million people in developing countries in 2000 and this value is projected to rise to 1.15 billion by 2025 [7]. Though the prevalence of hypertension is increasing in developing countries due attention has not been given regarding strategies for prevention and control since these countries are overwhelmed by health needs of communicable diseases [2,4].

Like other developing countries, Ethiopia is increasingly being affected by hypertension. For instance, as high as 31.5% and 28.9% in male and females, respectively in Addis Ababa were reported [8]. Another study reported 19.1% prevalence of hypertension among bank workers and teachers in Addis Ababa, with higher (22%) in men than in women (14.9%) in 2009 [9]. Two other studies reported 13.2% hypertension prevalence in southwest Ethiopia [10] and 28.3% hypertension prevalence in Gondar town [11]. In a more recent study conducted in Addis Ababa in 2014 the prevalence of hypertension found to be 25.0% with significantly higher prevalence of hypertension in males (30.2%) than females (21.2%) [12]. However, due attention has not been given to prevent and control hypertension though studies indicated hidden epidemic of hypertension in Ethiopian population.

The risk factors for increasing prevalence of hypertension include population growth, aging and easily modifiable risky behaviors, like unhealthy diet, harmful use of alcohol, smoking, lack of physical activity, overweight/obesity and longstanding stress [12,13]. Studies in Ethiopian populations have shown that the odds of developing hypertension is higher in male sex, advancing age, being overweight/obese, being physically inactive, high salt intake, family history of hypertension, and being urban dweller [8,10,11,14].

To the best of our knowledge, data on incidence of hypertension in Southern Ethiopia is scarce. Two studies reported, one hospital based and the other institution based, reported 18.8% overall prevalence of hypertension in diabetic and non-diabetic controls [15] and 19.7% crude prevalence of hypertension, respectively [16]. Therefore, this study aimed to assess the prevalence of hypertension and associated risk factors in Bona district, southern Ethiopia, at community level.

## Materials and methods

### Study setting and design

This is a cross-sectional community based study conducted on 1952 participants aged 25 years and above, in Bona district, South Ethiopia. Bona district (‘woreda’ in Ethiopia setting) is one of the 19 districts in Sidama zone, South Nations Nationalities and People’s state. The district has 20 villages (‘kebele’, the smallest administrative unit in Ethiopia). The data were collected from February to June 2016 from 2670 participants aged 15–110 years for diabetes prevalence study. In this study, data for participants of age ≥25 years were included in analysis.

Residents of the Bona district, who have lived at least half a year in the study area and who gave informed consent were included in the study. Pregnant women, severely ill patients at the time of data collection and individuals with fever, infection and congestive heart failure were excluded from the study.

### Sampling techniques

The sample size was estimated by taking 0.50 proportion since national prevalence for hypertension is lacking. The calculated sample size at 95% confidence interval and absolute precision (d) of 0.02 was 2401. Multistage sampling technique was applied to select study participants. From 20 villages of the district, 10 were selected by lottery method, and each village was allocated sample size proportional to the total households in each village. A constant number k was obtained by dividing the total household in each selected village by the sample size allotted to the respective village. Then, the first household was selected from each selected village randomly by lottery method, and subsequent households were selected by taking consecutive k^th^ households until the allotted sample size for each village was obtained. Only one participant was recruited into the study from a household, by using lottery method in households where there were more than one eligible individuals.

### Data collection

The data were collected by data collectors consisted of general practitioners and nurses who were able to speak and write the local language. The data collectors were given one day training about the study and the data collection process by the principal investigator. To ensure data quality, the questionnaire was standardized by 5% pretesting and random supervisions were done by investigators. The general practitioners made physical examination for clinical conditions for consented individuals before they were interviewed by trained nurses using structured questionnaire. The data were collected in three categories: socio-demographic information, previous history of hypertension and treatment, and family history of hypertension. After the interview, anthropometric parameters and blood pressure were measured for each participant.

Blood pressure was measured after at least 5 minutes of rest using an appropriate mercury sphygmomanometer and expressed in mmHg. Hypertension was defined based on WHO criteria, systolic blood pressure ≥140 mmHg or diastolic blood pressure ≥90mmHg or reported regular use of anti-hypertensive drug [2]. The measurements were taken two times and the average value was recorded.

Weight was measured to the nearest 0.1 kilogram (kg) using a person scale when the participants were in light indoor clothing and bare feet. Height was measured to the nearest 0.01 meter (m) by stadiometer when the participants were in erect position without shoes. Body mass index (BMI) was calculated by dividing weight in kilograms by square of height in meters. BMI < 18.0kg/m^2^ is taken as underweight, 18.0–24.9kg/m^2^ as normal, 25.0–29.9kg/m^2^ as overweight and ≥30kg/m^2^ as obese [17]. Waist circumference was measured to the nearest of 0.01 meter (m) by placing a tape meter horizontally, midway between the 12^th^ rib and the iliac crest on the mid-axillary line. Waist-to-height ratio (WHtR) was calculated by dividing waist circumference in meters by height in meters. Both male and female participants with WHtR ≥0.50 were considered as having central obesity based on suggestions from previous studies [18,19].

### Data analysis

Data were entered, cleaned, coded and analyzed by using SPSS for Windows version 20.0 (IBM, USA). The data were cleaned by using sort cases tool and whenever missing and/or unexpected values were identified, that value was checked in the filled hardcopy data collection questionnaire to correct data entry mistakes. Continuous variables were expressed in mean and standard deviation of the mean or median and inter-quartile range. Since the outcome variable was ordered categorical, we used multinomial logistic regression model to identify associated factors. Collinearity diagnostics was done, and there was no multicollinearity among the independent variables included in the model. An odds ratio (OR) with 95% CI was reported, while the level of significance was set at p < 0.05.

### Ethical approval

The research protocol was approved by the South Nations, Nationalities and Peoples’ Regional State Health Bureau Ethical Review Committee. Every effort was made to keep personal information in the research record private and confidential. Written informed consent was obtained from each participant before data collection.

## Result

### Characteristics of study participants

Data were collected from total of 2670 study participants, from which data of 1952 participants, aged 25 years and above, were included in the analysis. Table 1 depicts the socio-demographic and anthropometric characteristics of the study participants. More than half of the participants (53.4%) were male while the rest 46.6% were females. Most (96.8%) of the participants are of Sidama ethnic group and the rest 3.2% were from others. Most (85.0%) of them were married, followed by 10.1%, 3.1%, and 1.8% of widowed, single and divorced, respectively.

**Table 1:** Sociodemographic and anthropometric characteristics of study participants (N = 1952).

Variable		Frequency (%)	Median (Interquartile range)
Sex			
	Male	1043 (53.4)	
	Female	909 (46.6)	
Age			
	25–34 years	684 (35.0)	40 (30–54)
	35–44 years	491 (25.2)	
	45–54 years	294 (15.1)	
	55–64 years	214 (11.0)	
	≥65 years	269 (13.8)	
Marital Status			
	Single	60 (3.1)	
	Married	1659 (85.0)	
	Divorced	36 (1.8)	
	Didowed	197 (10.1)	
Ethnicity			
	Sidama	1890 (96.8)	
	Other	62 (3.2)	
Educational level			
	Illiterate	908 (46.5)	
	Elementary school	739 (37.9)	
	Secondary school	185 (9.5)	
	Postsecondary education	120 (6.1)	
Family size			
	Up to 5 members	646 (33.1)	6.7 (2.9)^¥^
	Greater than 5 members	1306 (66.9)	
Family income			
	500 birr or less	1138 (58.3)	475 (200–1000)
	Greater than 500 birr	814 (41.7)	
Body mass index			
	Overweight & obese	156 (8.0)	
	Normal	1545 (79.1)	20.93 (2.99)^¥^
	Underweight	251 (12.9)	
Waist-to-Height Ratio			
	High	612 (31.4)	0.48 (0.05)^¥^
	Normal	1340 (68.6)	
Family hypertension			
	Yes	131 (6.7)	
	No	1821 (93.3)	

^¥^Mean (Standard Deviation)

The median age of the study participants was 40 (30–54) years and more than one third (35.0%) of the participants were in the age range of 25–34 years, followed by 25.2%, 15.1%, 13.8%, and 11.0%, in the age range of 35–44, 45–54, ≥65, and 55–64 years, respectively. Regarding education level, 39.1% of the study participants had elementary education, followed by 35.5%, 19.1%, and 6.2% of illiterate, secondary, and postsecondary education, respectively.

The average family size is 6.7 (2.9), which is similar to family size commonly observed in developing countries. When grouped into two categories, 66.9% of the participants live in families of more than 5 members and the rest 33.1% live in families of five or less members. The rough median family income was 475.00 (200.00–1000.00) Birr and more than half (58.3%) of the participants reported to have monthly income of 500 or less Birr and 41.7% reported that they have more than 500 Birr monthly family income.

The mean BMI was 20.93 (2.99) kg/m^2^, with most of the study participants (79.1%) had normal body mass index (BMI), remarkable proportion (12.9%) of them were underweight and 8.0% of them were overweight and obese. Nearly one third (31.4%) of participants had central obesity, as measured by WHtR, and it was significantly higher in females (40.6%, P<0.001) compared to that in males (23.3%). Only 6.7% of the participants reported that they have family history of hypertension.

### Prevalence of hypertension and associated factors

The mean systolic and diastolic blood pressure, respectively were 117.0 (21.1) mmHg and 75.5 (13.3) mmHg. In our study hypertension was defined based on the WHO criteria, systolic blood pressure 140mmHg and above or diastolic blood pressure 90mmHg and above or reported regular use of anti-hypertensive drugs [2]. Out of the participants included in analysis, 21.8% had hypertension either by systolic or diastolic blood pressure, 12.3% in males and 9.4% in females. Out of the hypertensive group, 47.1% (200) were hypertensive both by systolic and diastolic blood pressure that constituted 10.2% of the total participants. From the hypertensive group, 27.3% and 25.6% were hypertensive by systolic and diastolic blood pressure alone, respectively. One hundred night five participants (45.9%) of the hypertensive participants were newly diagnosed. About 10.6% of the participants had prehypertension.

Identifying the predictors of hypertension is important for taking corrective measures to prevent the alarmingly increasing prevalence of hypertension in all populations. As indicated in Table 2, since the dependent variable is ordered categorical, we used multinomial logistic regression to identify predictors of hypertension. Being male (16.6%) is of higher risk of developing systolic hypertension than being female (15.7%), OR 1.37 (95% CI: 1.00, 1.89, P<0.050). Systolic hypertension was significantly increased progressively with age advancement that its prevalence in participants with age of 65 years and above (35.7%) is significantly higher than that in participants with age range of 25–34 years (7.5%), OR 6.21 (95% CI: 3.99, 9.68, P<0.001).

**Table 2:** Estimation of systolic hypertension and ordinal logistic regression analysis of factors associated with systolic blood pressure in study participants (N = 1952).

Variables		Systolic Blood Pressure(mmHg)				
		≤120 (N = 1440)	121–139 (N = 196) = 196)	≥140 (N = 316)	OR [95% CI]	P value
Sex						
	Male	754 (72.3%)	116 (11.1%)	173 (16.6%)	1.37 (1.00, 1.89)	<0.050
	Female	686 (75.5%)	80 (8.8%)	143 (15.7%)	1.00	
Age						
	≥ 65 years	150 (55.8%)	23 (8.6%)	96 (35.7%)	6.21 (3.99, 9.68)	<0.001
	55–64 years	145 (67.8%)	22 (10.3%)	47 (22.0%)	3.40 (2.11, 5.48)	<0.001
	45–54 years	202 (68.7%)	32 (10.9%)	60 (20.4%)	2.98 (1.93,4.60)	<0.001
	35–44 years	366 (74.5%)	63 (12.8%)	62 (12.6%)	1.79 (1.19, 2.70)	<0.005
	25–34 years	577 (84.4%)	56 (8.2%)	51 (7.5%)	1.00	
BMI						
	≥25kg/m^2^	87 (55.8%)	24 (15.4%)	45 (28.8%)	2.60 (1.43, 4.71)	<0.002
	18.0–24.9kg/m^2^	1156 (74.8%)	153 (9.9%)	236 (15.3%)	1.25 (0.83, 1.90)	
	<18.0kg/m^2^	197 (78.5%)	19 (7.6%)	35 (13.9%)	1.00	
WHtR						
	High	391 (63.9%)	75 (12.3%)	146 (23.9%)	1.83 (1.36, 2.45)	<0.0010
	Normal	1049 (78.3%)	121 (9.0%)	170 (12.7%)	1.00	
Educational level						
	Post-secondary	90 (75.0%)	16 (13.3%)	14 (11.7%)	0.95 (0.47, 1.93)	
	Secondary school	142 (76.8%)	24 (13.0%)	19 (10.3%)	0.69 (0.38, 1.24)	
	Primary school	568 (76.9%)	74 (10.0%)	97 (13.1%)	0.82 (0.58, 1.15)	
	Illiterate	640 (70.5%)	82 (9.0%)	186 (20.5%)	1.00	
Family size						
	>5 members	944 (72.3%)	143 (10.9%)	219 (16.8%)	1.03 (0.77, 1.38)	
	≤5 members	496 (76.8%)	53 (8.2%)	97 (15.0%)	1.00	
Family income						
	>500 birr	621 (76.3%)	85 (10.4%)	108 (13.3%)	0.72 (0.55, 0.96)	<0.022
	≤500 birr	819 (72.0%)	111 (9.8%)	208 (18.3%)	1.00	
Family HTN						
	Yes	94 (71.8%)4	11 (8.4%)	26 (19.8%)	1.43 (0.88, 2.33)	
	No	1346 (73.9%)	185 (10.2%)	290 (15.9%)	1.00	

WHtR–waist-to-height ratio, HTN–hypertension, BMI–body mass index, OR-odds ratio. The test is significant at α < 0.05.

The other predictor of systolic hypertension was BMI. The prevalence of systolic hypertension in participants with BMI ≥ 25 kg/m^2^ (28.8%) was significantly higher than that of participants with BMI 18.0–24.9kg/m^2^ (15.3%), OR 2.60 (95% CI: 1.43, 4.71, P<0.002). Nearly one third of the study participants had central obesity (as measured by WHtR ≥0.50) and the prevalence of systolic hypertension is significantly higher in participants with WHtR ≥0.5 (23.9%) compared to 12.7% of participants with WHtR <0.50, OR 1.83 (95% CI: 1.36, 2.45, P<0.001). Systolic hypertension was negatively associated with increased family income. The association of systolic hypertension with educational levels, family size, family income, and family history of hypertension was not statistically significant.

Like for systolic hypertension, multinomial logistic regression was done to identify risk factors for diastolic hypertension and results were depicted in Table 3. Diastolic hypertension was significantly associated with male sex, older age, high BMI, central obesity, and family hypertension history.

**Table 3:** Estimation of diastolic hypertension and ordinal logistic regression analysis of factors associated with systolic blood pressure in study participants (N = 1952).

Variables		Diastolic Blood Pressure(mmHg)				
		≤ 80 (N = 1632)	81–89 (N = 11) = 196)	≥90 (N = 309)	OR [95% CI]	P value
Sex						
	Male	855 (82.0%)	8 (0.8%)	180 (17.3%)	1.38 (1.01, 1.90)	<0.044
	Female	777 (85.5%)	3 (0.3%)	129 (14.2%)	1.00	
Age						
	≥ 65 years	192 (71.4%)	1 (0.4%)	76 (28.3%)	3.70 (2.40, 5.70)	<0.001
	55–64 years	176 (82.2%)	0 (0.0%)	38 (17.8%)	2.09 (1.29, 3.37)	<0.003
	45–54 years	237 (80.6%)	2 (0.7%)	55 (18.7%)	2.03 (1.33, 3.09)	<0.001
	35–44 years	413 (84.1%)	4 (0.8%)	74 (15.1%)	1.59 (1.09, 2.31)	<0.016
		614 (89.8%)	4 (0.6%)	66 (9.6%)	1.00	
BMI						
	≥25kg/m^2^	101 (64.7%)	2 (1.3%)	53 (34.0%)	2.52 (1.41, 4.48)	<0.002
	18.0–24.9kg/m^2^	1311 (84.9%)	9 (0.6%)	225 (14.6%)	1.12 (0.74, 1.72)	
	<18.0kg/m^2^	220 (87.6%)	0 (0.0%)	31 (12.4%)	1.00	
WHtR						
	High	459 (75.0%)	4 (0.7%)	149 (24.3%)	0.49 (0.12–0.85)	<0.009
	Normal	1173 (87.5%)	7 (0.5%)	160 (11.9%)	1.00	
Educational level						
	Post-secondary	97 (80.8%)	2 (1.7%)	21 (17.5%)	1.31 (0.69, 2.47)	
	Secondary school	155 (83.8%)	1 (0.5%)	29 (15.7%)	1.11 (0.65, 1.88)	
	Primary school	628 (85.0%)	5 (0.7%)	106 (14.3%)	0.99 (0.71, 1.39)	
	Illiterate	752 (82.8%)	3 (0.3%)	153 (16.9%)	1.00	
Family size						
	>5 members	1077 (82.5%)	6 (0.5%)	223 (17.1%)	1.20 (0.89, 1.60)	
	≤5 members	555 (85.9%)	5 (0.8%)	86 (13.3%)	1.00	
Family income						
	>500 birr	689 (84.6%)	5 (0.6%)	120 (14.7%)	0.83 (0.63, 1.09)	
	≤500 birr	943 (82.9%)	6 (0.5%)	189 (16.6%)	1.00	
Family HTN						
	Yes	99 (75.6%)	0 (0.0%)	32 (24.4%)	1.79 (1.15, 2.80)	<0.010
	No	1533 (84.2%)	11 (0.6%)	277 (15.2%)	1.00	

WHtR–waist-to-height ratio, HTN–hypertension, BMI–body mass index, OR–odds ratio coefficient, HTN—Hypertension. The test is significant at α < 0.05

Being male sex sets higher risk of developing diastolic hypertension in our study, OR 1.38 (95% CI: 1.01, 1.90, P<0.044). Diastolic hypertension increases with age advancement; in participants aged 65 years and above the risk of developing diastolic hypertension significantly increased, OR 3.70 (95% CI: 2.40, 5.70, P<0.001).

The other predictor of diastolic hypertension was high BMI in which its prevalence in participants with BMI ≥25kg/m^2^ (34.0%) is significantly higher compared to participants with normal BMI (12.4%), OR 2.52 (95% CI: 1.41, 4.48, P<0.002). Central obesity was measured by WHtR in our study and we found that participant with high WHtR have higher risk of developing diastolic hypertension, OR 1.88 (95% CI: 1.40, 2.51, P<0.001). The prevalence of diastolic hypertension was significantly higher in participants with family history of hypertension (24.4%) compared to participants without family history of hypertension (15.2%), OR 1.79 (95%: 1.15, 2.80, P<0.010).

The rest of socio-demographic and economic variables included in the regression model were not significantly associated with diastolic hypertension.

## Discussion

It has been well established that hypertension increases the risk of cardiovascular disease and mortality [1,2]. The detection, prevention, management and control of hypertension is insufficient in low income countries [20] in face of higher burden of hypertension in low income countries than in high income countries [21]. The findings of this study strengthens reports from previous studies [8,10–12,14,16] that hypertension is becoming a silent epidemic in the country and calling policymakers for timely intervention in terms of creating awareness, early detection and management. We estimated the magnitudes of systolic, diastolic and combined hypertension in rural setting. Out of 1952 participants, 425 (21.8%) had hypertension either by systolic or diastolic blood pressure from which 47.1% (200) had combined hypertension, indicating that hypertension may be an important public health concern in study area. The risk factors for both systolic and diastolic hypertension were being male sex, age advancement, high body mass index and central obesity. In addition, family history of hypertension is risk for high diastolic blood pressure.

The observed prevalence of hypertension, 21.8%, seems remarkably higher in rural setting. Such a high prevalence of hypertension may indicate the disorder became a hidden epidemic in the community. In addition, nearly half (45.9%) of the hypertensive participants were newly diagnosed, indicating that blood pressure checkup level is low in the studied population. The prevalence of hypertension in our study is similar to the prevalence in rural Kenya, 21.4% [22], urban Kenya, 22.8% [23], urban Malawi, 22.5% [24], and Durame town (southern Ethiopia), 22.4% [14]. The high prevalence of hypertension in our study, among other studies in Ethiopia and other African countries, may be due to age advancement, nutritional transition and changing lifestyle like physical inactivity. However, the prevalence of hypertension observed in our study is higher than reported from other studies in Ethiopia in urban settings and other African countries in rural settings; 13.2% in southwest Ethiopia that had included both rural and urban participants [10], 19.7% among workers of Hawassa University [16], 19.3% in rural Nigeria, and 14.5% in rural Malawi [24]. In contrary, the observed hypertension in our study is lower than that reported from Ethiopia and other African countries. For example, two studies in Addis Ababa, 30.3% by Tesfaye et al [8] and 25% by Abdissa et al [12], and 28.3% in Gondar town [11] reported higher prevalence of hypertension than ours. Moreover, the prevalence of hypertension in our study is lower than 23.6% [25] and 24.8% [26] prevalence of hypertension in rural Nigeria and 24.1% in rural and 32.9% in semi-urban settings of Ghana [27]. These discrepancies in prevalence of hypertension may be due to differences in socio-demographic settings, physical activity, nutritional status, economic status, ethnic groups, and age of participants included.

As observed in previous studies [8,9,12,28] hypertension was higher in males (12.3%) than in females (9.4%) in our study. Systolic hypertension as well as diastolic hypertension were significantly higher in males (16.6% systolic and 17.3% diastolic) than females (15.7% systolic and 14.2% diastolic), which is consistent with the previous reports in Ethiopia [8] and other African countries [29]. For instance, prevalence of systolic and diastolic hypertension in males (24.9% systolic and 21.8% diastolic), which is significantly higher than that in female (20.3% systolic and 18.0% diastolic) was reported in Addis Ababa [8]. The higher prevalence of hypertension in males than in females may be due to sex-difference in effect of genetic doses and hormones such as rennin-angiotensin-aldosterone system and gonadal hormones [30,31].

As expected, prevalence of systolic as well as diastolic hypertension increased progressively with age advancement, which leads to increased arterial stiffness. The highest prevalence of both systolic hypertension and diastolic hypertension were observed in age of 65 years and above, 35.7% (P<0.001) and 28.3% (P<0.001), respectively. This is in agreement to previous studies in Ethiopia [8,11,12,16] and elsewhere in other African countries [23,27]. In fact, the high prevalence of hypertension our study may due to age advancement since 39.8% of study participants were in age of 45 years and above. Therefore, it is advisable to design methods to promote blood pressure check up as age advances to ensure early detection and management of hypertension. Other studies also advice early detection and management of hypertension [11,22].

The other risk factor for development of hypertension was increased BMI, which is measure of body fat load. Both systolic hypertension (28.8%, P<0.002) and diastolic hypertension (34.0%, P<0.002) in participants with BMI ≥25.0 kg/m^2^ were significantly higher respectively compared to 15.3% and 14.6% prevalence in participants with BMI 18.0–24.9 kg/m^2^. Other studies in Ethiopia [8] and other African countries [32] reported similar finding. The prevalence of central obesity as measured by WHtR ≥0.5 (31.4%) is remarkably high in rural setting; with significantly higher prevalence in females (40.6%, χ^2^ = 67.5, P<0.001) compared to that in males (23.3%). High WHtR is positively and significantly associated with both systolic hypertension (23.9%, P<0.001) and diastolic hypertension (24.3%, P<0.009). Thus, it can be inferred that the observed high prevalence may be due to increased central obesity in the studied population. Previous studies reported that increased WHtR strongly predicted the probability of developing hypertension in both children and adults [33,34].

The association of hypertension with family size, educational level and family income is not significant. However, the prevalence of systolic hypertension is highest in illiterates and high family income is inversely associated with both systolic and diastolic hypertension. Family history of hypertension significantly increases the chance of developing diastolic hypertension in this study (OR 1.79 (95% CI: 1.15, 2.80), P<0.010), while the prevalence of systolic hypertension in participants with family history of hypertension (19.8%) is not significantly different from those without family history of hypertension (15.9%). Previous studies in different parts of the country [10,11] reported similar findings.

There were potential limitations of our study. One of the limitations is limited demographic and anthropometric measurements incorporated in our study. For example, to measure central obesity we used only waist-to-height ratio while using waist-to-hip ratio may better complement our findings. The other limitation of our study is dependence on only two blood pressure measurements on one instance to diagnose hypertension. Guidelines for diagnosis of hypertension recommend measurements of blood pressure on two or more instances [35]. Our study is limited to small locality so that it may not be generalized to the total population of Ethiopia.

## Conclusion

The overall prevalence of hypertension, 21.8%, is alarmingly high that it can be said that hypertension is becoming a silent epidemic in Ethiopia. This could be due to not making necessary adjustment in modifiable risk factors like physical inactivity, overweight and obesity and central obesity. Being male, age advancement, high BMI, central obesity and family history of hypertension are risk factors for both systolic and diastolic hypertension. Nationwide survey is needed to get the clear magnitude of hypertension so that early detection and management early detection and management strategies can be enforced.
